# Baseline Mentalization Predicts Depression and Alexithymia Following Brief Psychoanalytic Psychotherapy in Students Seeking Help at a University Clinic: A Pre–Post Study

**DOI:** 10.3390/ijerph23080956

**Published:** 2026-07-24

**Authors:** Maria Domenica Sauta, Giulia Raimondi, Giulia Tomaselli, Claudia Lanza, Cristiana Maria Avalle, Isabella Giulia Franzoi, Antonella Granieri

**Affiliations:** 1 Department of Psychology, University of Turin, via Verdi 10, 10123 Turin, Italy; mariadomenica.sauta@unito.it (M.D.S.); giulia.tomaselli@edu.unito.it (G.T.); claudia.lanza@unito.it (C.L.); cristiana.avalle@unito.it (C.M.A.); isabellagiulia.franzoi@unito.it (I.G.F.); antonella.granieri@unito.it (A.G.); 2 BeSSA Department, Wellbeing, Health and Environmental Sustainability, Sapienza University of Rome, via delle Fontanelle, 02100 Rieti, Italy

**Keywords:** mentalization, university students, brief psychotherapy, psychoanalytic psychotherapy, alexithymia, depression, student counseling center

## Abstract

**Highlights:**

**Public health relevance—How does this work relate to a public health issue?**

- University students represent a psychologically vulnerable population, frequently exposed to depressive symptoms, emotional dysregulation, and psychological distress during the transition to adulthood.
- Identifying clinically relevant psychological processes associated with mental health outcomes may facilitate early detection and support the development of preventive and treatment-oriented interventions within university psychological services.

**Public health significance—Why is this work of significance to public health?**

- Mentalization may represent a key clinical dimension for understanding individual differences in affect regulation, interpersonal functioning, and vulnerability to psychological distress among university students.
- The findings support the conceptualization of mentalization as a transdiagnostic process associated with multiple domains of psychological functioning and emotional adaptation in young adults.

**Public health implications—What are the key implications or messages for practitioners, policy makers and/or researchers in public health?**

- Assessing mentalization during intake and early clinical evaluation may help identify students at greater risk of severe emotional difficulties, maladaptive coping, and poorer treatment outcomes.
- University mental health services may benefit from psychotherapeutic interventions specifically aimed at strengthening reflective functioning, emotional awareness, affect regulation, and the capacity to process interpersonal experiences.

**Abstract:**

Mentalization is a core psychological function underlying emotional regulation and vulnerability to psychopathology. This pre–post naturalistic study examined whether baseline mentalization predicts depressive symptoms and alexithymia in university students undergoing brief psychoanalytic psychotherapy. A sample of 104 students was assessed at baseline (T0) and post-treatment (T1). Longitudinal path analysis showed that baseline mentalization significantly predicted both depressive symptoms and alexithymia at follow-up, controlling for their temporal stability. Lower mentalization at intake was associated with poorer outcomes at treatment. completion The model demonstrated good fit and explained a substantial proportion of variance in both outcomes. These findings suggest that mentalization functions as a transdiagnostic vulnerability factor influencing psychotherapy outcomes and highlight the clinical importance of assessing and targeting mentalization from the early stages of treatment.

## 1. Introduction

Among university students, depressive symptoms and difficulties in emotional awareness are highly prevalent and frequently co-occur as expressions of more pervasive forms of psychological distress. The transition to university represents a critical developmental phase for emerging adults, involving substantial psychological reorganization and complex developmental tasks, such as identity consolidation, exploration of new relational and social roles, and future-oriented decision-making [1,2,3]. At the same time, students are confronted with multiple stressors related to academic demands, high performance expectations, relocation, separation from family, and the need to establish new social and living arrangements while progressively achieving autonomy and economic independence [4,5,6]. These intertwined developmental and contextual challenges may significantly impact mental health [7,8], with evidence consistently showing higher levels of psychological distress and psychopathology in university students compared to their non-student peers [5,9]. In particular, university students show high rates of depression, anxiety, suicide risk, substance use, alcohol misuse, and other addictive behaviors, often reflecting broader forms of psychological distress [7,10,11,12,13]. Furthermore, such difficulties have been shown to negatively affect academic functioning and increase the risk of university dropout [10,14].

From a clinical perspective, these manifestations cannot be fully understood in terms of external stressors alone. They also require consideration of internal processes that organize subjective experience and contribute to somatopsychic balance, particularly emotion regulation, symbolization and reflective functioning. Within this framework, both theoretical and empirical literature suggest that mentalization, defined as the ability to understand one’s own and others’ mental states [15], represents a core psychological function underpinning affect regulation and vulnerability to psychopathology. From this perspective, mentalization may constitute a common factor underlying different expressions of emotional distress, making it a potentially relevant target for preventive and supportive interventions in help-seeking university students. In particular, a growing body of literature has documented a robust association between impaired mentalization and depressive symptomatology, suggesting that difficulties in symbolizing and making sense of emotional experience contribute to the development of depressive states [16,17]. In this view, reduced reflective functioning may limit the individual’s capacity to regulate affects and integrate internal experiences, thereby sustaining both the severity and persistence of depressive symptomatology [18].

Closely related to mentalization is alexithymia, conceptualized as a multidimensional construct involving three components: inability to identify emotions and distinguish them from bodily sensations, difficulty describing emotions, and externally oriented thinking [19,20,21]. From a psychoanalytic perspective, alexithymia can be understood as an expression of impaired symbolization, whereby emotional experiences fail to be adequately represented at a mental level and are instead experienced in undifferentiated or somatic forms. Accordingly, alexithymia has been associated with increased vulnerability to both mental and physical health problems [22,23,24]. The literature highlights a strong association between impaired mentalization and alexithymia, suggesting that deficits in reflective functioning hinder the capacity to recognize, elaborate, and regulate emotional states [19,25].

Although mentalization and alexithymia are closely related constructs, they are not considered interchangeable. Indeed, mentalization refers to a broader reflective capacity involving the ability to perceive, interpret, and represent mental states in oneself and others, including emotions, intentions, motivations, and the underlying meanings of psychological experiences [26,27,28]. Alexithymia, in contrast, represents a more specific difficulty in emotional processing, particularly involving the identification and differentiation of emotional states, the ability to describe feelings to others, and a tendency toward externally oriented thinking [29,30].

Recent evidence further supports the close but distinct relationship between these constructs, showing that alexithymia is associated with impaired mentalization across self-, informant-, and meta-perception perspectives, while still representing a specific dimension of emotional processing difficulties [19]. Although both constructs involve difficulties in understanding and processing emotional experiences, mentalization encompasses a wider capacity for interpreting internal and interpersonal mental states, whereas alexithymia primarily concerns deficits in emotional awareness, differentiation, and verbalization. Therefore, the present study does not assume that mentalization and alexithymia represent the same underlying phenomenon; rather, it hypothesizes that reduced mentalization may constitute a broader vulnerability factor associated with subsequent difficulties in emotional processing, including alexithymic features [28,31].

Regarding the relationship between mentalization and depression, the experience of continuous affective mirroring by the caregiver promotes the development of mentalization while also increasing the value attributed to one’s own emotions and the perception of emotions as meaningful and useful experiences. Accordingly, individuals with greater mentalizing abilities, particularly in their affective components, tend to show higher emotional awareness and more effective affect regulation, and are less vulnerable to depressive symptoms. Conversely, impairments in mentalization have been associated with increased vulnerability to the development and maintenance of depressive features [32].

Despite the growing body of literature linking mentalization to both depressive symptoms and alexithymia, most existing studies have relied on cross-sectional designs or have examined these constructs independently, without considering their potential shared underlying mechanism. To date, limited research has investigated the longitudinal role of mentalization as a shared underlying factor influencing multiple domains of psychological distress within a naturalistic clinical setting. Moreover, little is known about the extent to which baseline mentalization may prospectively predict psychological outcomes following brief psychotherapeutic interventions in help-seeking university students. Addressing these gaps, the present study adopts a longitudinal design to examine mentalization as a transdiagnostic vulnerability factor, simultaneously accounting for the temporal stability of depressive symptoms and alexithymia.

Specifically, in the present study, we hypothesized that mentalization would represent a strong longitudinal predictor of depressive symptoms and alexithymic traits in a sample of university students attending the Clinical Psychology and Psychotherapy Area of the “ResidenzAscolta” service [“Residence is Listening”]. This service is specifically designed for university students residing in residences managed by the Piedmont Regional Agency for the Right to University Education (EDiSU Piemonte), in northern Italy. In particular, we examined whether levels of mentalization assessed during the initial psychological consultation (T0) could be identified as a shared underlying factor associated with subsequent levels of depressive symptoms and alexithymic features at the end of a brief psychoanalytic psychotherapy intervention. Specifically, a parsimonious longitudinal path model was specified to test whether baseline mentalization was associated with later depressive symptoms and alexithymia, while accounting for the temporal stability of the constructs under investigation.

## 2. Materials and Methods

### 2.1. Study Design

This is a longitudinal, naturalistic study conducted within a university-based clinical psychology and psychotherapy service for students residing in EDISU Piemonte university residences.

### 2.2. Clinical Pathway

The ResidenzAscolta service provides free psychological support to university students. The service is structured as a stepped-care model designed to receive, assess, and, when indicated, provide psychological treatment for difficulties related to emotional well-being, interpersonal functioning, and academic adjustment.

**Figure 1:**
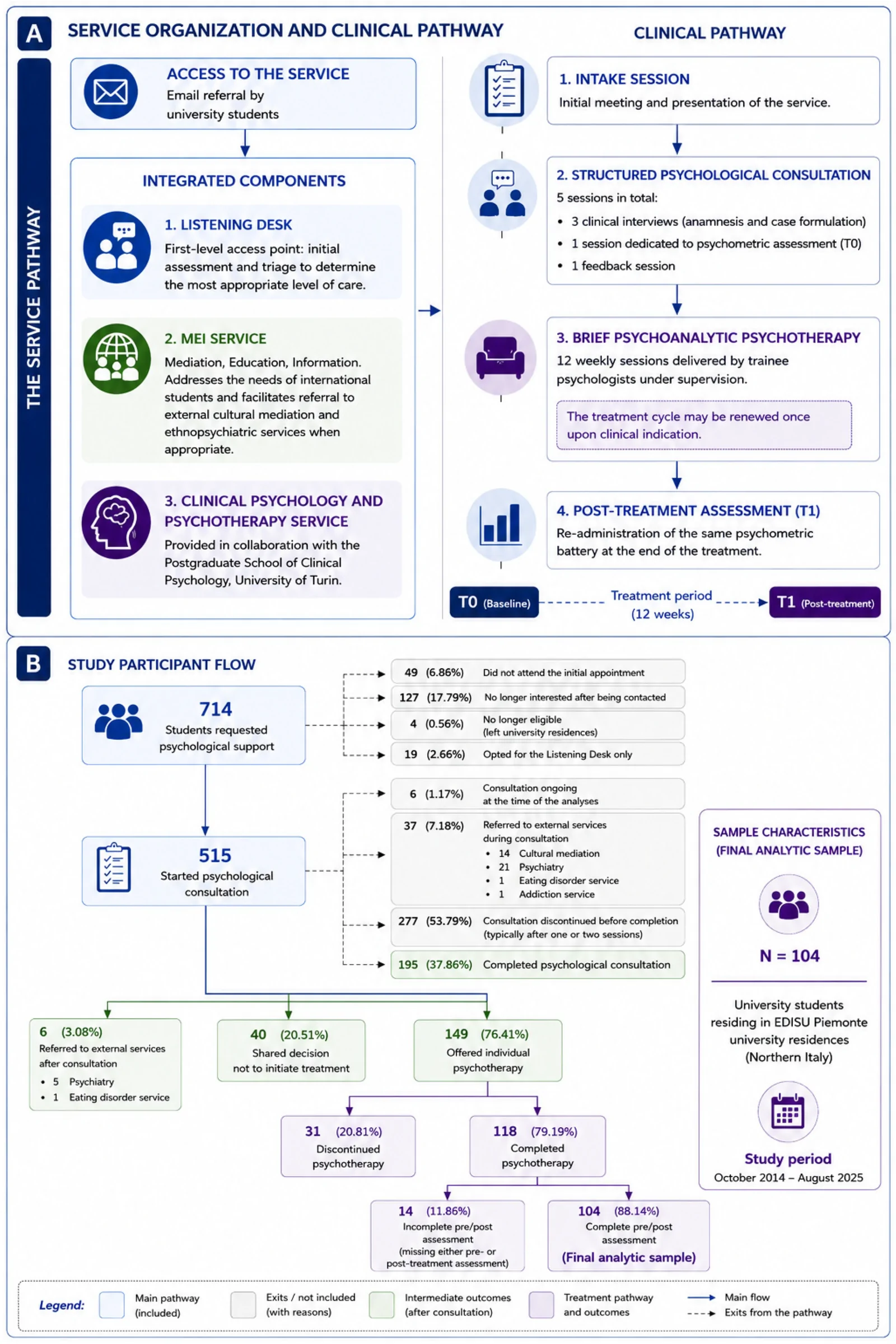
ResidenzAscolta Service organization and Participant Flow.

Access is initiated via email referral. The service includes three integrated components (see Figure 1):

- Listening Desk [Sportello di Ascolto]: first-level access point providing initial assessment and triage to determine the most appropriate level of care.
- MEI service [Mediation, Education, Information]: staffed by a psychologist and an anthropologist, this area primarily addresses the needs of international students and facilitates referral to external cultural mediation and ethnopsychiatric services when appropriate.
- Clinical Psychology and Psychotherapy service: delivered in collaboration with the Postgraduate School of Clinical Psychology, Department of Psychology, University of Turin.

Clinical activities within the latter service are delivered by licensed psychologists enrolled in the Postgraduate School of Clinical Psychology, under the supervision of a designated clinical tutor [CMA] and the scientific coordinator of the project [AG]. The clinical pathway consists of an initial intake session followed by a structured psychological consultation comprising five sessions: three clinical interviews focused on anamnesis and case formulation; one session dedicated to standardized psychometric assessment (T0); and one feedback session.

Following the consultation phase, patients may enter a brief psychoanalytic psychotherapy, consisting of 12 weekly sessions. The intervention did not follow a manualized psychoanalytic psychotherapy protocol. Rather, it was conducted within a psychoanalytic framework that informed clinicians’ listening, case formulation, and therapeutic interventions, while allowing flexibility according to each patient’s individual needs. In keeping with psychoanalytic practice, therapeutic interventions were not organized around predefined techniques or session-by-session structured procedures but emerged from the clinical material presented by each patient and from the evolving therapeutic relationship. Accordingly, the focus of treatment was not fixed in advance but was guided throughout the process by the psychological difficulties that appeared most clinically relevant and amenable to change within the time-limited format.

The same psychometric assessment administered at baseline (T0) is re-administered at treatment completion (T1). The treatment cycle may be renewed once upon clinical indication.

### 2.3. Participant Flow

As this was a naturalistic study conducted within a routine stepped-care psychological service, participant flow reflected routine clinical pathways. From October 2014 to August 2025, a total of 714 students requested access to the Clinical Psychology and Psychotherapy Unit. Of these, 49 (6.9%) did not attend the initial appointment, 127 (17.8%) declined participation after being contacted, 4 (0.6%) were no longer eligible because they had left the University Residence, and 19 (2.7%) opted to receive only brief counseling rather than entering the full clinical pathway. Consequently, 515 students initiated the consultation process.

At the time of data extraction, six participants were still undergoing the consultation phase and were therefore not eligible for subsequent analyses. Among the remaining 509 participants, 37 (7.3%) were referred to external services because this was considered the most appropriate clinical option (cultural mediation, psychiatric services, eating disorder services, or addiction services), 277 (54.4%) discontinued the consultation before its completion, and 195 (38.3%) completed it.

Following the consultation, treatment decisions were based on individualized clinical evaluation. Among the 195 participants who completed the consultation phase, six (3.1%) were referred to external specialist services because this was considered the most appropriate clinical option, 40 (20.5%) reached a shared decision with the clinician not to initiate psychological treatment, and 149 (76.4%) were offered individual psychotherapy.

Of the 149 participants who started psychotherapy, 31 (20.8%) discontinued treatment before completion, whereas 118 (79.2%) completed the intervention. Fourteen participants who completed psychotherapy were excluded from the pre–post analyses because of incomplete outcome assessment data. Complete pre- and post-treatment assessment data were available for 104 participants (14.6% of the original 714), who therefore constituted the final analytic sample (see Table 1 for descriptive statistics of the sample).

The study flow diagram (Figure 1) was created with the assistance of ChatGPT-5.5 (OpenAI) as a graphical drafting tool. The authors provided the source material, defined the diagram content and structure through iterative prompting, and critically reviewed and refined the final figure to ensure that it accurately represented the study methodology.

### 2.4. Measures

The full assessment battery included a broader set of validated self-report instruments that were refined over time for different clinical and research purposes. However, for the aims of the present study, only a selected subset of measures was considered: the Mentalization Questionnaire (MZQ), the Toronto Alexithymia Scale (TAS-20), and the Beck Depression Inventory-II (BDI-II), all administered at both time points (T0 and T1).

The MZQ [33,34] is a 15-item self-report measure assessing the capacity to represent and reflect upon one’s own and others’ mental states. Items are rated on a 5-point Likert scale (from 1 = “I agree” to 5 = “I disagree”), with higher scores reflecting poor mentalization ability. Internal consistency was good at both time points (T0: α = 0.829; T1: α = 0.827).

The TAS-20 [35,36] is a 20-item self-report instrument assessing alexithymia across three domains: difficulty in identifying feelings, difficulty in describing feelings, and externally oriented thinking. Items are rated on a 5-point Likert scale, and a total score is computed by summing all items. For the purpose of the present study, alexithymia was considered as a global construct; therefore, only the total score was used in all analyses. Internal consistency was good at both time points (T0: α = 0.821; T1: α = 0.851).

The BDI-II [37,38] is a 21-item self-report measure assessing depressive symptom severity over the past two weeks. Items are rated on a 4-point scale (0–3), with higher scores indicating greater severity. Internal consistency was excellent at both time points (T0: α = 0.907; T1: α = 0.910).

### 2.5. Ethical Considerations

The study was conducted in accordance with the Declaration of Helsinki. Ethical approval was obtained from the competent institutional Bioethics Committee of the University of Turin (protocol number: 162317, approved 19 April 2018). All participants provided written informed consent prior to participation in the study. Participation was voluntary, and no interference with access to clinical services occurred as a result of study participation. Data were collected and analyzed in anonymized form to ensure confidentiality and compliance with data protection regulations.

### 2.6. Statistical Analysis

To evaluate the potential impact of treatment attrition and missing outcome data, two additional baseline comparisons were performed. First, participants who completed psychotherapy were compared with those who discontinued treatment before completion. Second, participants with complete pre- and post-treatment assessment data were compared with those with incomplete outcome data. Independent-samples *t*-tests were used for continuous variables and Fisher’s exact tests for categorical variables. Analyses were performed using all available baseline data; therefore, sample sizes varied slightly across variables due to occasional missing baseline assessments.

A path analysis with observed-variable was conducted across two waves (T0–T1) in the sample of 104 participants with complete data on the variables included in the model. The model included autoregressive paths from baseline depressive symptoms (BDI_T0) to depressive symptoms at follow-up (BDI_T1) and from baseline alexithymia (TAS_T0) to alexithymia at follow-up (TAS_T1), in order to control for their temporal stability. Direct paths were specified from baseline mentalization (MZQ_T0) to both depressive symptoms and alexithymia at T1 (whilst controlling for their autoregressive paths). In addition, baseline depression and alexithymia were allowed to covary, as was baseline mentalization with both baseline symptom measures. This model was specified a priori and kept deliberately parsimonious, focusing on theoretically motivated associations. The following indices were used to assess model fit: the Root Mean Square Error of Approximation (RMSEA), with values below 0.05 indicating evidence of absolute fit [39,40]; the Comparative Fit Index (CFI), with values > 0.95 indicating good model fit [41]; the Standardized Root Mean square Residual (SRMR), with values < 0.08 indicating good fit [42]; and the chi-square (χ^2^) test, with *p*-values greater than 0.05 indicating an adequate fit to the data. Prior to path analysis, multicollinearity among baseline predictors (MZQ_T0, BDI_T0, and TAS_T0) was assessed by computing Variance Inflation Factors (VIFs) and Tolerance values. All analyses were performed with Jamovi (version 2.5.3.0) [43].

In addition, paired-samples two-tailed *t*-tests were conducted to examine mean-level changes between baseline (T0) and follow-up (T1) in Mentalization Questionnaire (MZQ), Toronto Alexithymia Scale (TAS-20), and Beck Depression Inventory-II (BDI-II) scores. Descriptive statistics, including means and standard deviations, were calculated for each variable at both assessment points.

## 3. Results

Baseline demographic and clinical characteristics were compared between participants who completed psychotherapy and those who discontinued treatment to evaluate potential attrition bias. No statistically significant differences emerged for age, gender, nationality, or baseline TAS, MZQ, and BDI scores (Table 2).

A second analysis compared participants with complete (n = 104) and incomplete (n = 14) outcome data. Likewise, no statistically significant baseline differences were observed between the two groups. Participants with incomplete outcome data tended to report lower baseline TAS scores; however, this difference did not reach statistical significance (*p* = 0.054). Because analyses were based on all available baseline data, the number of observations varied slightly across variables, particularly for the MZQ, for which baseline data were available for only two participants with incomplete outcome data.

**Figure 2:**
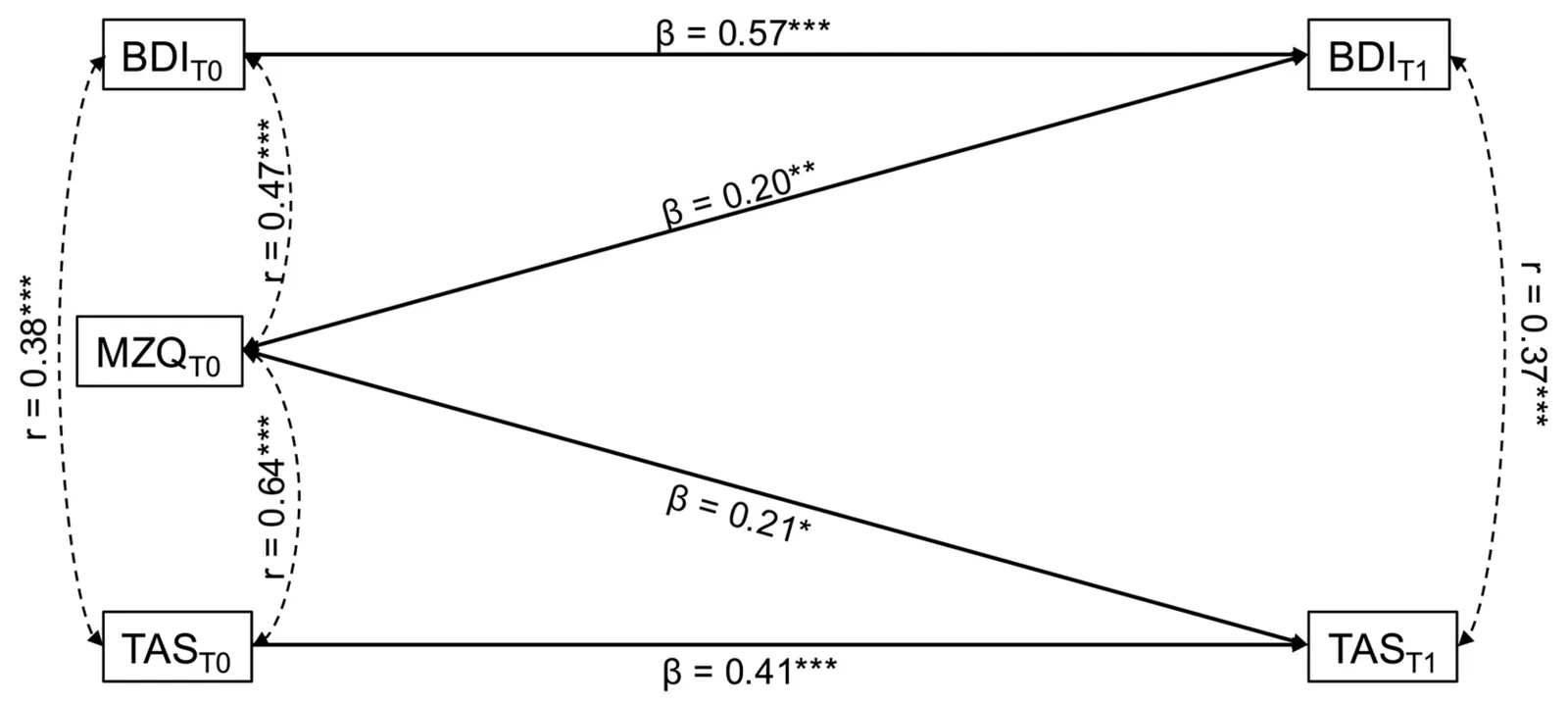
Path diagram of the posited model. *Note*. Standardized beta values are reported, as well as covariances among constructs. * *p* < 0.05; ** *p* < 0.01; *** *p* < 0.001.

Descriptive statistics for the key study variables, including Mentalization Questionnaire (MZQ), Toronto Alexithymia Scale (TAS-20), and Beck Depression Inventory-II (BDI-II) scores at baseline (T0) and post-treatment (T1), are reported in Table 3. In addition, paired-samples *t*-tests were conducted to descriptively examine mean-level changes over time. Results showed significant reductions from T0 to T1 across all psychological measures considered. Specifically, TAS-20 total scores significantly decreased from baseline (M = 53.9, SD = 12.4) to follow-up (M = 51.4, SD = 11.4), t (103) = 2.26, *p* = 0.026. Similarly, MZQ total scores significantly decreased from T0 (M = 33.5, SD = 10.8) to T1 (M = 31.5, SD = 10.2), t (103) = 2.32, *p* = 0.022. Finally, BDI-II scores also showed a significant reduction over time, decreasing from a mean of 20.7 (SD = 12.6) at baseline to 16.0 (SD = 10.6) at follow-up, t (103) = 5.12, *p* < 0.001. The model (see Figure 2) demonstrated good fit to the data (χ^2^ = 1.85, *p* = 0.396; CFI = 1.00; RMSEA = 0.00–95% CI [0.000–0.190]; SRMR = 0.027). As expected, both depressive symptoms and alexithymia showed substantial temporal stability, with baseline depression strongly predicting depressive symptoms at follow-up (β = 0.57, *p* < 0.001) and baseline alexithymia strongly predicting alexithymia at follow-up (β = 0.41, *p* < 0.001). Importantly, baseline mentalization significantly predicted both outcomes at T1, with lower mentalization at T0 predicted higher levels of depressive symptoms at T1 (β = 0.20, *p* = 0.010) as well as higher levels of alexithymia at T1 (β = 0.21, *p* = 0.042). The model explained 47.2% of the variance in depressive symptoms at T1 and 31.8% of the variance in alexithymia at T1.

Finally, multicollinearity among baseline predictors was ruled out. All VIF and Tolerance values were well below conventional thresholds (VIF < 10; Tolerance > 0.20), indicating no multicollinearity concerns (MZQ_T0: VIF = 1.90, Tolerance = 0.53; BDI_T0: VIF = 1.31, Tolerance = 0.76; TAS_T0: VIF = 1.73, Tolerance = 0.58).

## 4. Discussion

The present study investigated the relationship between mentalization, alexithymic traits, and depressive symptoms in a sample of university students undergoing a brief psychoanalytic psychotherapy consisting of 12 weekly sessions.

Baseline comparisons showed no evidence of systematic differences between psychotherapy completers and dropouts or between participants with complete and incomplete outcome data. These findings suggest that attrition and missing outcome data were unlikely to have substantially biased the observed pre–post results, although residual bias related to unmeasured factors cannot be excluded in a naturalistic clinical study.

For what concerns the sample of 114 students with complete outcome data, the findings indicate that mentalization assessed at the initial consultation (T0) significantly predicts levels of alexithymia and depressive symptoms at the end of treatment (T1).

More specifically, lower baseline mentalization was associated with higher levels of both alexithymic traits and depressive symptomatology at follow-up. Although the observed effects were modest in magnitude, they remain relevant because they emerged after accounting for the strong temporal stability of the outcomes (i.e., autoregressive paths), thus reflecting associations over and above baseline levels. These results support the view of mentalization as a core vulnerability factor, linked to multiple domains of psychological distress.

From a psychoanalytic perspective, mentalization is not conceptualized as a fixed trait but as a dynamic and multilayered capacity, closely related to the ability to symbolize emotional experience. Its relative stability under conditions of stress has been proposed to support emotional regulation and maintenance of a coherent sense of self [44]. Conversely, reduced mentalizing capacities may be associated with difficulties in reflective functioning, leading to breakdowns in emotional regulation and symbolization processes [45,46].

Within this framework, university students seeking psychological support who present with vulnerabilities in mentalization may experience greater difficulty in processing emotional states and, consequently, show increased susceptibility to depressive symptomatology [8,47,48]. However, the present findings should not be interpreted as evidence of a direct causal pathway, but rather as indicating a longitudinal association between baseline reflective functioning and subsequent psychological outcomes.

A particularly significant finding of the present study is that baseline mentalization was associated with both alexithymia and depressive symptoms at the end of a brief psychoanalytic psychotherapy. Importantly, this finding does not imply that the intervention was ineffective in reducing symptoms or improving psychological functioning; rather, such outcomes fall outside the scope of the present analyses and warrant further investigation. Rather, the results suggest that, independent of treatment effects, the level of mentalization at baseline exerts a robust predictive influence on post-treatment outcomes.

One possible interpretation is that individuals with more impaired reflective functioning at intake may experience greater clinical difficulties and higher levels of psychopathological symptoms even after completing therapy. It is also plausible that such impairments limit the capacity to symbolize and elaborate emotional experience within the therapeutic relationship, thereby constraining the therapeutic process itself.

This interpretation is consistent with the literature describing a vicious cycle between impaired mentalization, alexithymia, and depressive symptomatology [47]. Reduced reflective functioning contributes to depressive experiences, which in turn further undermine mentalization capacities. In parallel, when emotional and bodily signals cannot be transformed into symbolic and meaningful representations, they tend to remain at a somatic and undifferentiated level, fostering emotional dysregulation and somatization processes, which are central to alexithymia [49,50].

Although the longitudinal design allows for the examination of temporal associations, it does not permit causal inference. Nevertheless, these findings contribute to the understanding of mentalization as a potential early marker of vulnerability across multiple domains of psychological distress. From a clinical standpoint, these findings underscore the importance of addressing mentalization processes from the very beginning of treatment, particularly in brief psychoanalytic settings.

More broadly, the results highlight the need for university-based clinical services to integrate interventions specifically aimed at enhancing reflective functioning, symbolization, and emotional regulation. Strengthening these capacities may not only reduce depressive symptoms and alexithymic traits but also improve engagement with psychotherapy and prevent the consolidation of more severe or chronic psychopathological conditions.

### Limitations and Future Directions

Several methodological limitations should be acknowledged.

First, the sample consisted of university students seeking help at a clinical psychology and psychotherapy service, which may limit the generalizability of the findings. An additional limitation concerns the sample size, which may have limited the statistical power to detect small or more complex effects, particularly in longitudinal analyses.

While this enhances ecological validity, as the data derive from routine clinical practice, it also implies that results may not extend to student populations who do not seek psychological treatment.

An additional limitation concerns participant attrition and missing outcome data, which are inherent challenges in naturalistic clinical studies. Because treatment decisions and referrals reflected routine clinical practice rather than a standardized research protocol, some degree of selection bias cannot be entirely excluded. However, baseline comparisons showed no statistically significant differences between psychotherapy completers and treatment dropouts or between participants with complete and incomplete outcome data, suggesting that the pre–post findings were not substantially influenced by systematic differences in the observed baseline demographic or clinical characteristics. Nevertheless, the possibility of bias related to unmeasured variables cannot be ruled out.

Furthermore, the study relied exclusively on self-report measures, which may be affected by response biases and, importantly, by the very capacities under investigation. Constructs such as mentalization and alexithymia are intrinsically linked to the ability to access, represent, and reflect upon internal states. Consequently, impairments in reflective functioning may also compromise the accuracy of self-report data.

In addition, although the longitudinal design allows for the examination of temporal associations, the study included only two assessment points (T0 and T1). This limits the ability to capture intra-individual fluctuations over the course of treatment and precludes analysis of potentially bidirectional relationships among variables. Future studies should include multiple measurement points and session-level assessments to better model change processes and within-therapy dynamics.

Moreover, future research should incorporate process-oriented measures to clarify how mentalization is activated, supported, or hindered within psychotherapy, particularly within the therapeutic relationship.

A further limitation is the absence of a control or comparison group. All participants received brief psychoanalytic psychotherapy, which does not allow disentangling treatment effects from spontaneous symptom change or external influences. Furthermore, potential attrition between T0 and T1 was not explicitly addressed.

Finally, the model did not include potential confounding variables such as personality traits, attachment patterns, or prior treatment history, which may influence both mentalization and clinical outcomes. Their inclusion would allow for a more comprehensive and clinically nuanced understanding of the observed associations.

## 5. Clinical Implications

The present findings have several clinically relevant implications for university-based mental health services and brief psychoanalytic interventions.

First, the results highlight the importance of assessing mentalization at intake. Baseline reflective functioning appears to be a clinically meaningful predictor of treatment outcomes, suggesting that initial evaluation is not only diagnostic but also prognostic in nature, identifying students at risk for poorer post-treatment outcomes in terms of depressive symptoms and alexithymic traits.

Second, the findings underscore the importance of actively supporting mentalization processes within psychotherapy. Interventions aimed at enhancing the capacity to identify, differentiate, and symbolize emotional states—particularly within the here-and-now of the therapeutic relationship—may be crucial for improving clinical outcomes. This is especially relevant in brief therapy formats, where time constraints require a focused engagement with core psychological functions.

Third, the results suggest that individual differences in mentalization may influence responsiveness to brief psychoanalytic psychotherapy. Students with more impaired reflective functioning at baseline may encounter greater difficulty in using the therapeutic setting to elaborate emotional experience. This highlights the importance of tailoring treatment planning to patients’ reflective capacities; while longer or more structured interventions may be beneficial in some cases, such options are not always feasible in university service settings.

More broadly, these findings support the integration of mentalization-informed approaches within university clinical services. Given the high prevalence of emotional distress among students, interventions that explicitly target reflective functioning and emotional awareness may contribute not only to symptom reduction, but also to strengthening underlying psychological capacities, thereby promoting more stable and enduring mental health outcomes.

Finally, mentalization may represent a transdiagnostic clinical marker useful for both assessment and intervention across a range of psychological difficulties. Future clinical protocols may benefit from systematically incorporating the evaluation and enhancement of reflective functioning as a core component of care for university students.

## 6. Conclusions

This naturalistic longitudinal study provides evidence that baseline mentalization is associated with subsequent depressive symptoms and alexithymia in university students undergoing brief psychoanalytic psychotherapy. Specifically, lower levels of mentalization at baseline predicted poorer psychological outcomes at the end of treatment, even after accounting for the temporal stability of depression and alexithymia, supporting the role of mentalization as a transdiagnostic psychological process linked to multiple domains of emotional functioning. From a clinical perspective, the results suggest that assessing mentalization during the initial evaluation may help identify students who are more vulnerable to poorer treatment outcomes and who may require greater therapeutic attention to reflective functioning and emotional processing. Within university mental health services, integrating mentalization-informed assessment and intervention strategies may contribute to improving the effectiveness of psychological care and to promoting more adaptive emotional functioning.

## Figures and Tables

**Table 1:** Descriptive statistics of the sample (n = 104).

Variables	n/M	%/(SD)
**Age M; (SD)**	22.09	(2.30)
**Biological sex n;%**		
Female	59	56.7%
Male	45	43.3%
**Nationality n;%**		
Italian	93	89.4%
Other	11	10.6%
**University degree n;%**		
Bachelor’s degree	55	59.1%
Master’s degree	34	36.6%
Single-cycle degree	4	4.3%
**Previous psychological visits n;%**		
Yes	38	41.3%
No	54	58.7%
**Previous psychotherapy n;%**		
Yes	26	28.3%
No	66	71.7%
**Previous psychopharmacological therapy n;%**		
Yes	15	16.5%
No	76	83.5%
**Current psychopharmacological therapy n;%**		
Yes	3	5.7%
No	50	94.3%
**Medical comorbidities n;%**		
Yes	6	20.0%
No	24	80.0%

***Note***. n = count; M = mean; % = percentage; SD = Standard Deviation. Percentages are calculated based on available data. N may vary across variables due to missing data.

**Table 2:** Baseline comparisons according to psychotherapy completion and outcome data availability.

Variable	Psychotherapy Dropouts	Psychotherapy Completers	*p*
**Age, years**	22.77 ± 3.23 (n = 31)	22.78 ± 2.28 (n = 117)	0.994
**Female, n (%)**	19 (61.3)	68 (58.1)	0.839 ^a^
**Italian nationality, n (%)**	28 (90.3)	106 (90.6)	1.000 ^a^
**TAS T0**	49.67 ± 10.95 (n = 30)	53.08 ± 12.16 (n = 118)	0.143
**MZQ T0**	34.25 ± 9.34 (n = 20)	33.29 ± 10.83 (n = 106)	0.685
**BDI T0**	22.10 ± 10.84 (n = 30)	20.12 ± 12.31 (n = 118)	0.389
	**Incomplete Outcome Data**	**Complete Outcome Data**	***p***
**Age, years**	21.86 ± 1.88 (n = 14)	22.90 ± 2.31 (n = 104)	0.108
**Female, n (%)**	9 (64.3)	59 (56.7)	0.775 ^a^
**Italian nationality, n (%)**	13 (92.9)	93 (89.4)	1.000 ^a^
**TAS T0**	47.21 ± 8.22 (n = 14)	53.87 ± 12.41 (n = 104)	0.054
**MZQ T0**	22.00 ± 7.07 (n = 2)	33.51 ± 10.80 (n = 104)	0.137
**BDI T0**	15.79 ± 9.27 (n = 14)	20.70 ± 12.59 (n = 104)	0.162

***Note***. Continuous variables are presented as mean ± standard deviation. Sample sizes vary across variables because analyses were performed using all available baseline data. ^a^ Fisher’s exact test.

**Table 3:** Paired-samples *t*-test results of the variables of the model.

Variables	T0	T1	*p* Value
**Mentalization M/(SD)**	33.5/(10.8)	31.5/(10.2)	0.022
**Depression M/(SD)**	20.7/(12.6)	16/(10.6)	<0.001
**Alexithymia M/(SD)**	53.9/(12.4)	51.4/(11.4)	0.026

***Note***. M = mean;% = SD = Standard Deviation.

## Data Availability

The original contributions presented in this study are included in the article. Further inquiries can be directed to the corresponding author.
